# Electrochemical Deposition and Etching of Quasi-Two-Dimensional Periodic Membrane Structure

**DOI:** 10.3390/molecules29081775

**Published:** 2024-04-13

**Authors:** Binbin Yao, Yongsheng Xu, Benzhuo Lou, Yinbo Fan, Erwei Wang

**Affiliations:** School of Physics and Telecommunication Engineering, Shaanxi University of Technology, Hanzhong 723000, China; yaobb@snut.edu.cn (B.Y.); loubenzhuo@126.com (B.L.); fanyb@snut.edu.cn (Y.F.); erweiwang@126.com (E.W.)

**Keywords:** electrochemical deposition, wave-shaped progressive growth, thin film, etching, micro-nano wires array

## Abstract

In this paper, two experimental procedures are reported, namely electro-deposition in the ultrathin liquid layer and chemical micro-etching. Firstly, a large area quasi-two-dimensional periodic membrane with adjustable density is deposited on a Si substrate driven by half-sinusoidal voltage, which is composed of raised ridges and a membrane between the ridges. The smaller the voltage frequency is, the larger the ridge distance is. The height of a raised ridge changes synchronously with the amplitude. The grain density distribution of membrane and raised ridge is uneven; the two structures change alternately, which is closely related to the change of growth voltage and copper ion concentration during deposition. The structural characteristics of membrane provide favorable conditions for micro-etching; stable etching speed and microscope real-time monitoring are the keys to achieve accurate etching. In the chemical micro-etching process, the membrane between ridges is removed, retaining the raised ridges, thus a large scale ordered micro-nano wires array with lateral growth was obtained. This method is simple and controllable, can be applied to a variety of substrates, and is the best choice for designing and preparing new functional materials. This experiment provides a basis for the extension of this method.

## 1. Introduction

With the progress of science and technology, the research and application of new functional micro-nano materials are constantly emerging, bringing new possibilities to various fields. Micro-nano materials refer to multi-scale composite materials in the scale of micron to nanometer, which have mesoscopic characteristics and span the macro and micro worlds on the length scale, showing excellent quantum size effects, surface and interface effects of micro-nano materials, and have potential application value in many fields. Compared with other micro-nano metals, copper based micro-nano materials have attracted much attention because of their abundant raw materials, low price and excellent physical and chemical properties which are different from macroscopic materials, which are often used in electrocatalysts, electronic circuits, sensors and surface-enhanced Raman scattering [1,2,3,4]. In particular, they provide the possibility for the construction of non-enzymatic glucose sensors due to their direct electrooxidation effect on some small molecular organic compounds [5,6], which would alleviate the problems of enzymatic biosensors, such as lack of stability, low repeatability, poor thermal and chemical stability, and sensitivity to pH, temperature and humidity [7,8]. As such, it is likely to become a fourth-generation glucose sensor for future analytical applications. It is well known that Ag conductive thin film has been widely used in the design and manufacture of electronic devices, but its large-scale application is limited by high price and limited reserve. In contrast, copper-based conductive thin films have an electrical conductivity comparable to silver, high-cost performance, and are easy to combine with flexible substrates, which have a wide application prospect in the field of next-generation flexible electronic products, and have attracted much attention [9,10,11,12,13,14]. It is considered a good substitute for silver-based conductive films. In addition, with the development of micro-nano processing and preparation technology, the study of thin film materials has shifted from smooth to periodic structure (hole array, linear array, disk array, etc.), and such materials can be controlled by changing structural parameters (array period, linear array height, etc.). In particular, quasi-two-dimensional materials have two dimensions, but the thickness is between a few nanometers and hundreds of nanometers. It has been found that quasi-two-dimensional periodic thin films have excellent performances in many aspects, such as magneto-optical properties, which has led to the becoming a hot spot in terms of new micro-nano structured functional materials. Another class of copper-based materials that is getting a lot of attention is one-dimensional micro-nano arrays. Ordered arrays arranged by combinations of a single micro-nano wire are important one-dimensional structures with a high surface area to volume ratio and excellent sensitivity and activity, which is very sensitive to species attached to the surface [15,16,17,18,19,20,21]. 

Customization and tailoring of dimension and size is an important goal in the field of materials science. Researchers have also developed a variety of methods to try to achieve the design and tailoring of material structures. Clearly, the precise control of size and morphology can be achieved through a micromachining process based on lithography technology [22,23,24]. However, this method has high requirements on process equipment and operating environment, and the cost is expensive. Compared with the top-down method, the bottom-up method is a relatively large and complex structural system formed by self-assembly of smaller structural units through weak interaction [25,26,27]. This method does not depend on precise equipment and has low cost, but it is difficult to achieve strict control of size and morphology. At present, template-assisted electrodeposition is considered as an effective method to prepare regular arrays because of the existence of uniformly distributed holes with adjustable density [28,29,30]. Unfortunately, it is difficult for nanowire arrays to be independent due to template constraints, and removing the template will result in the orderly disappearance of the long range between nanowire arrays. Nanoscale 3D printing has opened up a surprising world, and precise control of the feedback mechanism for the motion of the printed nozzle is a key element of success, otherwise it would quickly clog up, so printing extremely small metal structures using tiny nozzles has proven difficult [31,32,33]. It has long been expected that a micro-nano structure preparation process can fill the gap between the “top-down” and “bottom-up” technologies.

Recently, Chen et al. deposited U-shaped magnetic periodic array films on silicon grating with high aspect ratio surface morphology in a frozen electrolyte driven by square pulse. After etching, a set of separated vertical U-shaped micro-nano wires were obtained, and a low cost, high efficiency, high precision and large area controllable micro-nano structured array was realized [34]. This opens a new horizon for the design and synthesis of micro-nano structured periodic films and arrays. The construction of an ultra-thin liquid layer is a key problem in this technology. In the solute separation process of the frozen electrolyte, a structure of separate layers is formed between the two insulating substrates, which are similar to the sandwich structure; the ultra-thin liquid layer is above the base and below the ice–water mixed liquid layer. The ultra-thin liquid layer provides a quasi-two-dimensional growth space with a thickness in the range of 100 nanometers, which can inhibit ion diffusion and convection and reduce the branching of sedimentary structures [35]. Driven by the voltage between the anode and the cathode, the ions spread out from the cathode to the anode, like a wave-shaped progressive growth. The precise control of the material structure is realized by the change of voltage; the large-scale periodic thin films with regular micro-nano structure are synthesized. For the etching process, controlling time and speed, regardless of the method used, is also a crucial issue. In short, obtaining highly ordered and controllable periodic films and arrays by electrodeposition combined with a simple etching process is an ingenious design. At present, there is little research on the preparation of micro and nano structures using this technology; the technology is not well developed yet and needs to be perfected to realize popularization. 

Here, we plan to use a two-step method, ultra-thin liquid layer electrodeposition combined with wet etching to synthesize copper-based micro-nano periodic films and linear arrays. Considering the application prospect of copper-based materials, monocrystalline silicon substrate, which is mainly used in integrated circuits, transistors, solar cells and other fields is selected, which matches with copper-based materials. As is well known, wet etching has low cost, strong adaptability, and relatively high selection, but the etching rate is not easy to control due to isotropy, and it is difficult to achieve accurate etching [36]. To solve this problem, dynamic tracking of the microscope and temperature control was added during the etching process to ensure accuracy. The distance, height, and width of the ridges depend on the pulse signal of the deposition process. We choose a typical half-sinusoidal signal with a single frequency component to synthesize a copper-based micro-nano periodic structure. This experiment has expanded the preparation field of copper-based micro-nano structural materials and popularized the preparation technology.

## 2. Results and Discussion

**Figure 1:**
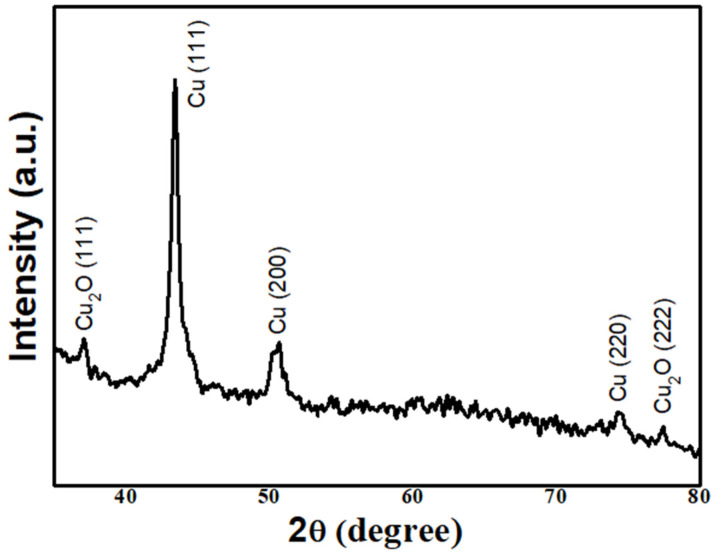
Representative XRD patterns of periodic film.

Figure 1 shows the XRD pattern of the periodic film, and the crystal structure of the deposited sample has been determined. The diffraction peaks are marked, and the positions of the three diffraction peaks are consistent with the JCPDS card (No. 04-0836) of copper, and are indexed with the (111), (200), and (220) planes of the cubic phase. The others are consistent with the JCPDS card (No. 65-3288) of cuprous oxide and are indexed with the (111) and (222) planes of the cubic phase. This indicates that the sediment is a composite structure of copper and cuprous oxide. It can also be seen that the intensity of cuprous oxide is lower than that of copper, indicating that the Cu content is much higher than Cu_2_O. According to the FWHM of the strongest peak of the diffraction peaks, the crystallite sizes of two components were calculated by Scherrer’s formula. The FWHM of 43.4° is about 0.5°, the crystallite size of Cu is about 17 nm; the FWHM of 37.1° is about 0.4°, and the crystallite size of Cu_2_O is about 21 nm. The crystallite sizes of the two components are basically the same, so the microstructure of the material should be uniform.

**Figure 2:**
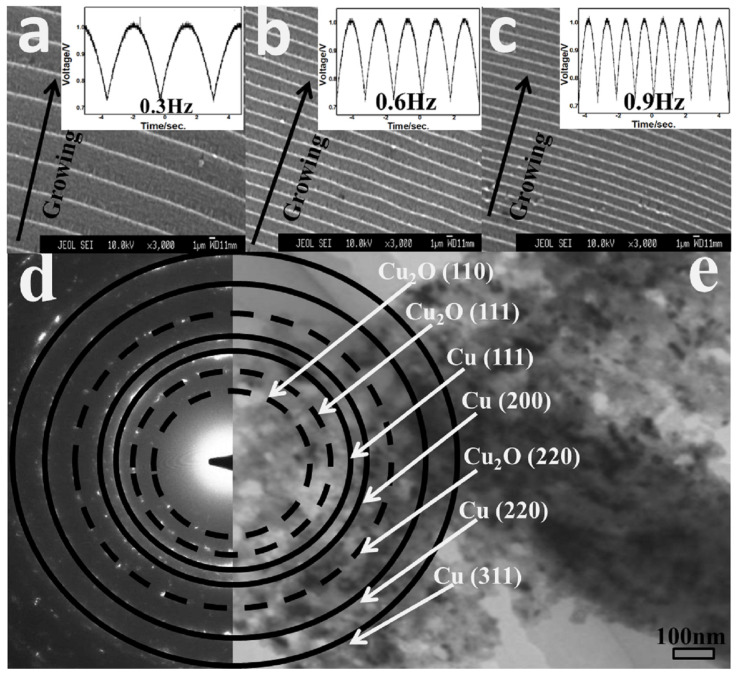
(**a**–**c**) SEM images of periodic structures growing on silicon substrates at half-sinusoidal voltages of different frequencies. (**d**,**e**) Representative electron diffraction and TEM image of periodic film.

Figure 2a–c shows the SEM images of samples 1–3, and the growth conditions are as described in the experiment section. The sediment was obtained under the drive of half sinusoidal pulse voltage; the insets in the right of Figure 2a–c are the images of voltage waveform. The deposited product presents a periodic thin film structure driven by a periodically changing voltage, and each period is composed of a raised ridge and membrane between ridges. It can be clearly seen that the ridge is bright and solid; the concave part is dark and thin. And, the period width is uniform and decreasing with increasing frequency. The growth direction is perpendicular to the electrodes, which is from cathode to anode. The raised ridge is the transverse growth, which is different from the growth pattern of conventional micro-nano wires (growing lengthwise along the axis). A more vivid metaphor for the growth process of the whole periodic structure is to push forward like a wave. The raised ridge corresponds to a wave crest, and the concave part is relatively gentle. 

Figure 2d shows the representative electron diffraction of periodic film (sample 2), the pattern is polycrystalline rings corresponding to Cu (111), Cu (200), Cu (220), Cu (311), Cu_2_O (110), Cu_2_O (111), and Cu_2_O (220). This further confirms that the sediment is composed of copper and cuprous oxide nanocrystals, which is consistent with the XRD results. Figure 2e shows a TEM image of periodic film (sample 2), which shows the structural characteristics. The high-density area corresponds to the bright and solid ridges in Figure 2a–c; the low-density areas on both sides connected to the high-density areas correspond to the dark and thin concave parts shown in Figure 2a–c. The raised ridge is about 400 nm in diameter and varies with voltage frequency. From the characteristics of the microstructure, it can be inferred that although the high-density area is narrow, the corresponding growth rate is slow, while the deposition rate is relatively fast for the low-density areas.

**Figure 3:**
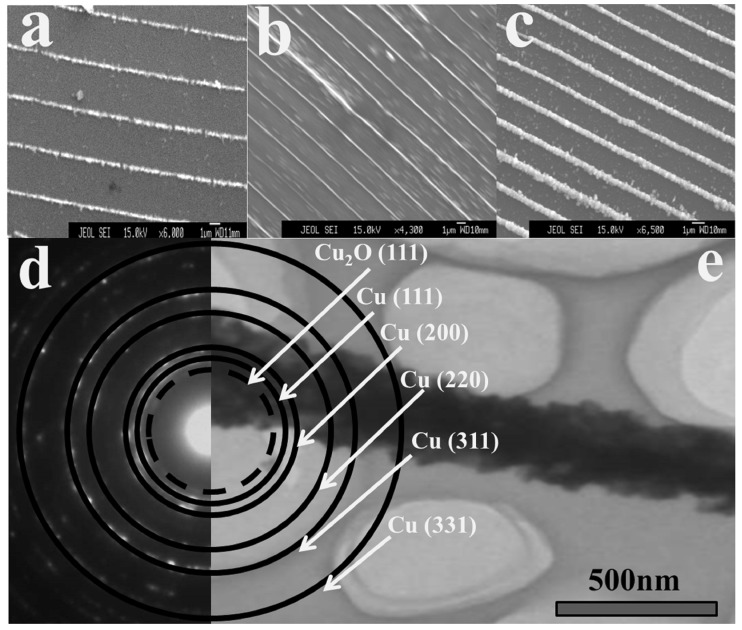
(**a**–**c**) SEM images of nanowires array under different frequencies. (**d**,**e**) Representative electron diffraction and TEM image of nanowires array.

According to the characteristics of the periodic structure, the chemical etching method is used to etch away the concave area of low density and retain the ridge of high density. Thus, highly ordered large-scale micro-nano wires can be obtained. The SEM images after etching are shown in Figure 3a–c, and the voltage frequency corresponds to that in Figure 2a–c. After fine etching, the low-density areas between ridges are removed. Figure 3d shows the representative electron diffraction of micro-nano wires; the pattern is polycrystalline rings corresponding to Cu (111), Cu (200), Cu (220), Cu (311), Cu (331), and Cu_2_O (111). This confirms that the wires are composed of copper and cuprous oxide nanocrystals. This indicates that chemical etching only changes the morphology of the sediment but does not change the crystal structure. Figure 3e shows a TEM image of a single wire, indicating that only the solid ridges of high density are retained after etching, and the low-density areas on both sides are completely removed. The diameter of the ridges is about 400 nm, and their size is also regulated by the frequency of the pulse voltage. The boundary on both sides of the ridges is not smooth due to the uneven density distribution, but the ridges are parallel to each other. A simple and controllable method is used to successfully carve large scale micro-nano wires; these wires are the transverse growth, which is perpendicular to the axis.

**Figure 4:**
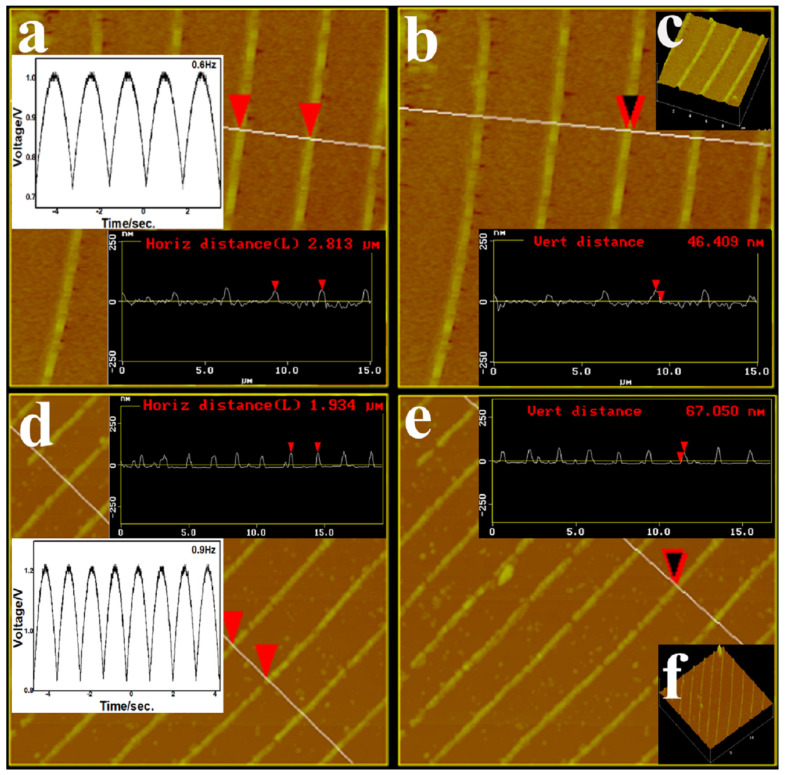
(**a**–**c**) AFM surface section images and three-dimensional surface image of periodic structure; (**d**–**f**) AFM surface section images and three-dimensional surface image of nanowires array. Note: The triangular cursor in the figure represents the selected measurement interval.

It is easy to understand that the morphology of periodic structure completely depends on the amplitude and frequency of the pulse voltage. In this study, the surface structure of periodic films was analyzed in depth by using the three-dimensional surface map provided by AFM. In Figure 4, the amplitudes of the periodic structure and the micro-nano wires array are 0.28 V and 0.36 V, and the frequencies are 0.6 Hz and 0.9 Hz, respectively. Figure 4a–c shows the AFM surface section images and three-dimensional surface image of periodic structure, the surface roughness is different, and the areas of high density are raised ridges. The ridges protrude from the surface, which are connected by membranes. It can be seen in Figure 4a,b that the distance between adjacent ridges is about 2.8 μm, and the ridge bulge height is about 46 nm. Figure 4d–f shows the AFM surface section images and three-dimensional surface image of the micro-nano wires array. Only the ridges are retained after etching, and the region between the ridges becomes smooth, resulting in the micro-nano wires array. The distance between adjacent ridges is about 1.9 μm, and the ridge bulge height is about 67 nm. It shows that the larger the frequency, the smaller the period width, and the larger the voltage amplitude, the higher the raised ridge. The surface distribution is concave and convex, and the density is not uniform, which is conducive to the engraving of patterns and the acquisition of an ordered array.

Periodic membranous structures are deposited in a quasi-two-dimensional growth space with a thickness in the range of 100 nanometers, which generally occurs according to the following process. Driven by the pulse voltage, Cu^2+^ in the ultra-thin liquid layer moves to the cathode, where electrons are reduced to Cu atoms and deposited on the cathode. At the same time, due to the loss of electrons, the Cu atoms of the anode are oxidized into Cu^2+^, which is continuously added to the ultra-thin liquid layer, so that the Cu^2+^ in the liquid layer maintains a dynamic balance on the whole, to ensure the continuous deposition of Cu in the cathode. It is worth noting that the minimum pulse voltage must satisfy the deposition potential of copper ions given by the Nernst equation, which is ~0.34 V.

**Figure 5:**
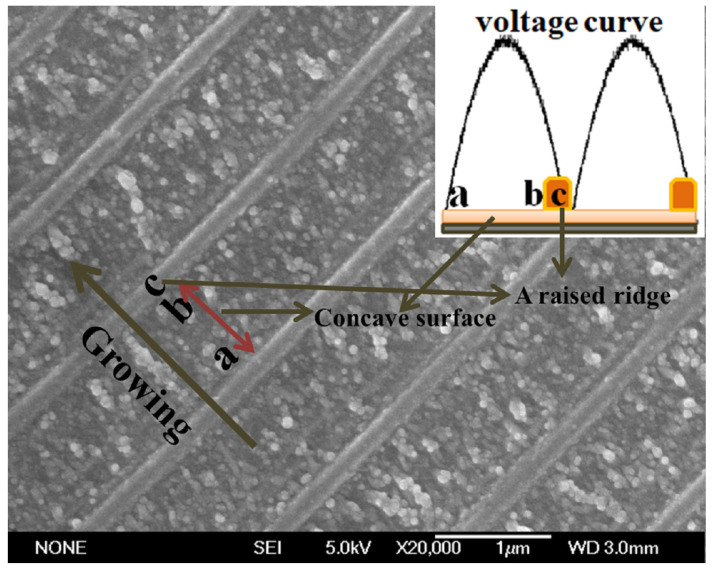
Growth principle illustration combined with high resolution TEM image and voltage curve.

In Figure 5, a periodic structure is marked with three parts, a, b, and c, along the growth direction; the a-b segment corresponds to the membrane of the periodic structure, and the c segment corresponds to the raised ridges. From high resolution SEM images, it can be seen that the grains of a-b accumulate linearly along the growth direction, with the density ranging from low to high. The particle density is highest in part c, and the grains accumulate into a raised ridge. From the perspective of dynamics, the growth rate of the a-region with lower density is faster; the growth rate of the dense b-region becomes slower; and the densest c-region has the slowest growth rate. From the perspective of one cycle of voltage transformation, the ridge region accounts for about one-third of the whole cycle. This growth phenomenon of the periodic membrane structure can be attributed to the delayed change of voltage and copper ion concentration at the growth front, which hinders ion migration [37]. To be specific, the change in Cu ion concentration near the growth interface always lags behind the change in electrode potential, so the deposition rate presents a periodic change, forming a periodic growth pattern [34,37]. When the electrolyte concentration is too low, a high resistance is formed between the electrodes. Electrolyte concentration affects the resistance between electrodes, so that the potential difference between electrodes changes synchronously with the actual voltage. When the actual voltage changes in the form of half sine, the corresponding cathode potential also changes. At the same time, with the change in electric field potential, copper ions move to the cathode, resulting in the change in the concentration of copper ions near the cathode. However, the ion migration rate is fixed, and the increase in ion concentration at the growth front always lags behind the change of actual voltage.

Initially, the actual voltage begins to rise, and the ion concentration is lowest near the cathode, where a large number of electrons accumulate. The voltage continues to increase, but the ion concentration always lags behind in relation to the charge consumed. In order to trap ions, any nucleation near the cathode will cause precipitates to protrude in the electrolyte, leading to the localized growth jetting. The linear and rapid growth forms a low-density zone, as shown in Figure 5. When the actual voltage reaches the maximum, the ion concentration fails to reach the maximum. When the actual voltage drops, the number of migrating ions is much greater than the number of reducing ions. This will cause some of the migrating ions to be reduced and the remaining ions to remain at the front of the sediment. So, the ion concentration at the front end of the growth is composed of residual ion concentration and migratory ion concentration. As the actual voltage continues to drop, the remaining ions begin to accumulate and the ion concentration begins to go up, leading to more nucleation and a lot of linear growth, eventually forming a slightly denser region, which is the b zone in Figure 5. When the actual voltage continues to drop, the ion concentration reaches the maximum, the high ion concentration will lead to the overall retardation of the growth pattern, and a large amount of nucleation rapidly accumulates, forming a high-density ridge perpendicular to the growth direction. The actual voltage continues to drop, reducing the supply of migrating ions, and thus, the ion concentration becomes lower. When the voltage returns to the minimum, the ion concentration also falls back to the minimum. The voltage goes up again, and the next cycle begins. The whole process pushes forward from cathode to anode like a wave. Finally, a quasi-two-dimensional periodic structure with alternating membrane and raised ridges is formed. This process is similar to the dynamic process of pulsed electrodeposition involved in other studies [34,37].

According to the surface analysis of AFM, the density and height of the membrane and ridge are different. We present an ingenious idea based on the characteristics of the sedimentary structure, to attempt to easily obtain a highly ordered and controllable micro-nano wires array by electrodeposition combined with a simple wet etching process. The micro-nano wire arrays carved by this method have the characteristics of transverse growth, are parallel to each other, have equal spacing, etc. The method is not bound by the template, it is simple, low cost, and can be produced at a large-scale. In the etching process, a mixture of KHSO_5_ and H_2_SO_4_ was used as an etching agent; the etching rate is uniform and controllable, and the chemical properties are stable. H_2_SO_4_ plays a very important role in the etching reaction, participating in the etching reaction and stabilizing the etching rate. The main etching reactions are as follows:

H_2_SO_4_ + KHSO_5_ + Cu = CuSO_4_ + KHSO_4_ + H_2_O

2H_2_SO_4_ + KHSO_5_ + Cu_2_O = 2CuSO_4_ + KHSO_4_ + 2H_2_O

In the process of chemical reaction, the addition of H_2_SO_4_ leads to the increase in HSO_4_^−^ content, which effectively inhibits the occurrence of forward reaction and stabilizes the etching rate. In addition, the etching process is controlled using a circulating water bath refrigeration system. The activity of particles and the reaction rate are also low when the temperature is low, so as to prolong the reaction time and improve the controllability. High controllability is convenient to observe the dynamic process of etching under the optical microscope, control the reaction time, and achieve accurate etching. The structural characteristics of the sediment and the etching conditions worked together to ensure that the lateral growth micro-nano wires array was carved out.

## 3. Materials and Methods

**Figure 6:**
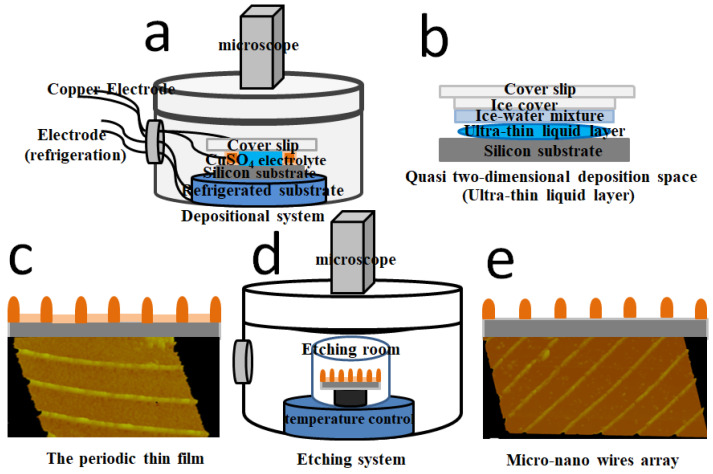
(**a**) The experimental system. (**b**) The quasi-two-dimensional deposition space. (**c**) The schematic diagram of the periodic structures of deposition. (**d**) The etching system. (**e**) The schematic diagram of linear periodic structures.

The main process of the experiment is shown in Figure 6. (a) The experimental system mainly includes the following parts: circulating water bath system, special adiabatic growth chamber, optical microscope, and camera device. (b) The construction of quasi-two-dimensional sedimentary space comprising the preparation of the deposited solution and the growth electrode, and the construction of the growth substrate and the ultra-thin liquid layer. First, CuSO_4_·5H_2_O with 99.99% purity was mixed with ultra-pure water to form 50 mmol/L electrolyte, and then evenly stirred with a magnetic agitator, pH value is about 4.0. Second, a fine copper wire with a diameter of 50 μm was selected, the surface was sanded gently to remove the insulating paint on its surface, and then it was soaked in alcohol and washed with ultra-pure water. Third, the silicon slice was cut into 2 cm × 2 cm square small pieces, put in acid, alkali solution, or alkaline hydrogen peroxide first to remove the ionic adsorption impurities, and then immersed in aqua regia or acid hydrogen peroxide to remove the remaining ionic impurities and atomic impurities. Finally, it was rinsed with deionized water and dried. Fourth, the treated silicon wafer was used as the substrate, and the copper wire, as the cathode and anode, was placed on the silicon substrate in parallel, the distance between the two electrodes was about 1.5 cm. The configured CuSO_4_ solution was added between the two copper wires, and the cover glass was placed on it. A semi-closed system was constructed in the growth chamber. The copper wires are connected with the external potential through the growth chamber, and the temperature in the growth chamber is controlled to be 2 °C using the circulating water device. The CuSO_4_ solution gradually becomes frozen, the solute continuously precipitates between the ice and the substrate, thus forming an ultra-thin liquid layer. The space of the CuSO_4_ electrolyte changes from three- to quasi two-dimensional, creating a sandwich space. In the process of solute separation, the current is controlled at about 2 mA. (c) Under the action of an applied pulse voltage, the depositing starts from the cathode and grows progressively towards the anode, forming a periodic film which is a quasi-two-dimensional structure with a film thickness of less than 100 nanometers, as shown in Figure 6c. The dynamic process of growth is like advancing waves. In this paper, the voltage amplitude of samples 1–3 is 0.28 V (ranging from 0.72 to 1.0), the corresponding frequencies are 0.3 Hz, 0.6 Hz, and 0.9 Hz, respectively; the amplitudes of the periodic structure (sample 4) and the micro-nano wires array (sample 5) are 0.28 V and 0.36 V, and the frequencies are 0.6 Hz and 0.9 Hz, respectively. (d) The precipitate is then placed in an etch mixture of 0.5 g/L KHSO_5_ and 1% H_2_SO_4_, and the circulating water bath controls the temperature at 2 °C. The etching process was observed with an optical microscope, and the etched samples were taken out after about 240 s, and then repeatedly cleaned with ultra-pure water and dried. (e) Etching removes the membrane between ridges and leaves the raised parts, thus forming the micro-nano wires array.

Field emission scanning electron microscopy (SEM), transmission electron microscopy (TEM), and atomic force microscopy (AFM) were used to characterize the morphology, microstructure, and surface structure of Cu_2_O/Cu micro-nano periodic structure. The sample composition was detected by X-ray diffractometer (XRD).

## 4. Conclusions

In this paper, a series of ordered quasi-two-dimensional micro-nano structures are deposited by regulating the frequency and amplitude of the voltage. The micro-nano structures are composed of raised ridges and interridge membranes. The morphology and structure characterization of the samples show that the voltage frequency is large; the periodic oscillation width is narrow; the voltage amplitude is great; and the elevation of the ridge is large. Combined with the change in the characteristics of growth voltage and copper ion concentration, the growth mechanism of the periodic membrane structure was analyzed. Then, a one-dimensional ordered micro-nano wires array is carved by etching method. By controlling the etching speed and dynamic monitoring, we ensure that the film between the ridges is completely removed, and the raised ridges are retained. It should be pointed out that the structural characteristics of sediments provide favorable conditions for micro-nano wires array etching. This technique is a preparation process of transverse growth wires array, and the samples are highly ordered and controllable. This method can be used to carve a variety of metals and their oxides. This experiment has expanded the preparation field of copper-based micro-nano structural materials and popularized the preparation technology.

## Data Availability

The data presented in this study are available on request from the corresponding author. The data are not publicly available due to the data management of the research group.

## References

[1] Zhang P., Sui Y., Xiao G., Wang Y., Wang C., Liu B., Zou G., Zou B. (2013). Facile fabrication of faceted copper nanocrystals with high catalytic activity for p-nitrophenol reduction. J. Mater. Chem. A.

[2] Han M., Yuan D., Liu S., Bao J., Dai Z., Zhu J. (2012). Facile synthesis of porous copper nanobelts and their catalytic performance. Mater. Res. Bull..

[3] Zhou X., Guo W., Zhu Y., Peng P. (2020). The laser writing of highly conductive and anti-oxidative copper structures in liquid. Nanoscale.

[4] Zhang B., Li W., Jiu J., Yang Y., Jing J., Suganuma K., Li C.-F. (2019). Large-scale and galvanic replacement free synthesis of Cu@Ag core-shell nanowires for flexible electronics. Inorg. Chem..

[5] Si P., Huang Y., Wang T., Ma J. (2013). Nanomaterials for electrochemical non-enzymatic glucose biosensors. RSC Adv..

[6] Wang G., He X., Wang L., Gu A., Huang Y., Fang B., Geng B., Zhang X. (2012). Non-enzymatic electrochemical sensing of glucose. Microchim. Acta.

[7] Tsai T., Heckert G., Neves L., Tan Y., Kao D.-Y., Harrison R.G., Resasco D.E., Schmidtke D.W. (2009). Adsorption of Glucose Oxidase onto Single-Walled Carbon Nanotubes and Its Application in Layer-By-Layer Biosensors. Anal. Chem..

[8] Chen S., Hai X., Chen X., Wang J. (2014). In Situ Growth of Silver Nanoparticles on Graphene Quantum Dots for Ultrasensitive Colorimetric Detection of H_2_O_2_ and Glucose. Anal. Chem..

[9] Zhang W., Zhou Y., Ding Y., Song L., Yuan Q., Zhao W., Xu C., Wei J., Li M., Ji H. (2022). Sintering mechanism of size-controllable Cu-Ag core-shell nanoparticles for flexible conductive film with high conductivity, antioxidation, and electrochemical migration resistance. Appl. Surf. Sci..

[10] Rahman M., Lu Z., Kwon K. (2018). Green laser sintering of copper oxide (CuO) nano particle (NP) film to form Cu conductive lines. AIP Adv..

[11] Li Z., Chang S., Khuje S., Ren S. (2021). Recent Advancement of Emerging Nano Copper-Based Printable Flexible Hybrid Electronics. ACS Nano.

[12] Cheng W., Lee M., Yasuda K., Song J.-M. (2022). Plasma-Modified PI Substrate for Highly Reliable Laser-Sintered Copper Films Using Cu_2_O Nanoparticles. Nanomaterials.

[13] Tomotoshi D., Kawasaki H. (2020). Surface and Interface Designs in Copper-Based Conductive Inks for Printed/Flexible Electronics. Nanomaterials.

[14] Li J., Wang Y., Tian H., Qi D., Wang R. (2020). Wood functional modification based on deposition of nanometer copper film by magnetron sputtering. For. Prod. J..

[15] Chang Y., Lin T., Wu Y., Fan S., Lee Y., Lai Y. (2019). Surfactant-assisted galvanic synthesis and growth characteristics of copper nanowires. Inorg. Chem. Front..

[16] Wang X., Yin X., Lai X., Liu Y. (2019). Magnetism, stability and electronic properties of a novel one-dimensional infinite monatomic copper wire: A density functional study. New J. Chem..

[17] Maji N., Chakraborty J. (2019). Gram-Scale Green Synthesis of Copper Nanowire Powder for Nanofluid Applications. ACS Sustain. Chem. Eng..

[18] Zhang T., Daneshvar F., Wang S., Sue H.-J. (2019). Synthesis of oxidation-resistant electrochemical-active copper nanowires using phenylenediamine isomers. Mater. Des..

[19] Ranjana M., Ramesh V., Satheesh Babu T., Kumar D.V.R. (2019). Sophorolipid induced hydrothermal synthesis of Cu nanowires and its modulating effect on Cu nanostructures. Nano-Struct. Nano-Objects.

[20] Ganapathi A., Swaminathan P., Neelakantan L. (2019). Anodic Aluminum Oxide Template Assisted Synthesis of Copper Nanowires using a Galvanic Displacement Process for Electrochemical Denitrification. ACS Appl. Nano Mater..

[21] Wang Y., Zhu Y., Chen J., Zeng Y. (2012). Amperometric biosensor based on 3D ordered freestanding porous Pt nanowire array electrode. Nanoscale.

[22] Isaacoff B., Brown K. (2017). Progress in top-down control of bottom-up assembly. Nano Lett..

[23] Kumar N., Salehiyan R., Chauke V., Botlhoko O., Setshedi K., Scriba M., Masukume M., Ray S.S. (2021). Top-down synthesis of graphene: A comprehensive review. FlatChem.

[24] Abid N., Khan A., Shujait S., Chaudhary K., Ikram M., Imran M., Haider J., Khan M., Khan Q., Maqbool M. (2022). Synthesis of nanomaterials using various top-down and bottom-up approaches, influencing factors, advantages, and disadvantages: A review. Adv. Colloid Interface Sci..

[25] Li F., Wang P., Huang X., Young D., Wang H., Braunstein P., Lang J. (2019). Large-Scale, Bottom-Up Synthesis of Binary Metal–Organic Framework Nanosheets for Efficient Water Oxidation. Angew. Chem..

[26] Pormehr A., Niyaifar M., Hasanpour A., Kheirdoust H., Elansary M., Niazi H. (2022). On the structural and magnetic properties of SbxY_3_-xFe_5_O_12_ nanostructures synthesized by bottom-up and top-down methods. J. Alloys Compd..

[27] Yang S., Wang Q., Zhao H., Liu D. (2022). Bottom-up synthesis of MOF-derived magnetic Fe-Ce bimetal oxide with ultrahigh phosphate adsorption performance. Chem. Eng. J..

[28] Guo B., Tian L., Xie W., Batool A., Xie G., Xiang Q., Jan S.U., Boddula R., Gong J.R. (2018). Vertically aligned porous organic semiconductor nanorod array photoanodes for efficient charge utilization. Nano Lett..

[29] Gupta J., Arya S., Verma S., Singh A., Sharma A., Singh B., Prerna, Sharma R. (2019). Performance of template-assisted electrodeposited Copper/Cobalt bilayered nanowires as an efficient glucose and Uric acid senor. Mater. Chem. Phys..

[30] Galindo A., Reyes-Rodríguez J., Botez C., Moreno M., Ponce A. (2022). Towards three-dimensional nanoarchitectures: Highly ordered bi-layer assembly of tailored magnetic nanowires array via template-assisted electrodeposition. Mater. Adv..

[31] Cheng L., Shoma Suresh K., He H., Rajput R.S., Feng Q., Ramesh S., Wang Y., Krishnan S., Ostrovidov S., Camci-Unal G. (2021). 3D Printing of Micro- and Nanoscale Bone Substitutes: A Review on Technical and Translational Perspectives. Int. J. Nanomed..

[32] Gu D., Chen H., Dai D., Ma C., Zhang H., Lin K., Xi L., Zhao T., Hong C., Gasser A. (2020). Carbon Nanotubes Enabled Laser 3D Printing of High-Performance Titanium with Highly Concentrated Reinforcement. Science.

[33] Ghosh K., Pumera M. (2021). Free-standing electrochemically coated MoSx based 3D-printed nanocarbon electrode for solid-state supercapacitor application. Nanoscale.

[34] Chen F., Yang Z.H., Li J.N., Jia F., Wang F., Zhao D., Peng R.-W., Wang M. (2022). Formation of magnetic nanowire arrays by cooperative lateral growth. Sci. Adv..

[35] Chen F., Li J., Yu F., Zhao D., Wang F., Chen Y., Peng R.W., Wang M. (2016). Construction of 3D metallic nanostructures on an arbitrarily shaped substrate. Adv. Mater..

[36] Horikiri F., Fukuhara N., Ohta H., Asai N., Narita Y., Yoshida T., Mishima T., Toguchi M., Miwa K., Sato T. (2019). Simple wet-etching technology for GaN using an electrodeless photo-assisted electrochemical reaction with a luminous array film as the UV source. Appl. Phys. Express.

[37] Cui G.L., Zhang P.H., Chen L., Wang X.L., Li J., Shi C., Wang D. (2017). Highly sensitive H_2_S sensors based on Cu_2_O/Co_3_O_4_ nano/microstructure heteroarrays at and below room temperature. Sci. Rep..

